# ‘What effect do safety culture interventions have on health care workers in hospital settings?’ A systematic review of the international literature

**DOI:** 10.12688/hrbopenres.13576.1

**Published:** 2022-07-01

**Authors:** Mairéad Finn, Lisa Mellon, Aisling Walsh, Niall O'Brien, David J. Williams, Natasha Rafter, Siobhán E. McCarthy

**Affiliations:** 1 Graduate School of Healthcare Management, RCSI University of Medicine and Health Sciences, Dublin, Ireland; 2 Department of Public Health and Epidemiology, RCSI University of Medicine and Health Sciences, Dublin, Ireland; 3 Library Services, RCSI University of Medicine and Health Sciences, Dublin, Ireland; 4 Department of Geriatric and Stroke Medicine, RCSI University of Medicine and Health Sciences, Dublin, Ireland

**Keywords:** patient safety, safety culture, hospital setting, healthcare worker, management

## Abstract

Introduction: Interventions designed to improve safety culture in hospitals foster organisational environments that prevent patient safety events and support organisational and staff learning when events do occur. A safety culture supports the required health workforce behaviours and norms that enable safe patient care, and the well-being of patients and staff. The impact of safety culture interventions on staff perceptions of safety culture and patient outcomes has been established. To-date, however, there is no common understanding of what staff outcomes are associated with interventions to improve safety culture and what staff outcomes should be measured.

Objectives: The study seeks to examine the effect of safety culture interventions on staff in hospital settings, globally. The research questions are: 1) what effects do interventions to improve safety culture have on staff? 2) What intervention features, safety culture domains or other factors explain these effects? 3) What staff outcomes and experiences are identified?

Methods and Analysis: A mixed methods systematic review will be conducted in accordance with the Preferred Reporting Items for Systematic Reviews and Meta-Analyses (PRISMA) guidelines. Searches will be conducted using the electronic databases of MEDLINE, EMBASE, CINAHL, Health Business Elite, and Scopus. Returns will be screened in Covidence according to inclusion and exclusion criteria. The mixed-methods appraisal tool (MMAT) will be used as a quality assessment tool. The Cochrane Collaboration’s tool for assessing risk of bias in randomised trials and non-randomised studies of interventions will be employed to verify bias. Synthesis will follow the Joanna Briggs Institute methodological guidance for mixed methods reviews, which recommends a convergent approach to synthesis and integration.

Discussion: This systematic review will contribute to the international evidence on how interventions to improve safety culture may support staff outcomes and how such interventions may be appropriately designed and implemented.

## Introduction

In 2000, the seminal report by the Institute of Medicine, *To Err is Human,* estimated over 90,000 patient deaths from medical error related to both system failure and poor organisational culture ^1^. Since that time the incidence of patient safety events have remained high globally, due in part to the increasing complexity of healthcare and multi-morbidities ^2, 3^. *To Err is Human* recommended the concept of a patient safety culture to support the health workforce to provide safer care ^1, 4^. A major focus is on the reduction and mitigation of patient safety events, building a culture of reporting and learning from events, and supporting those affected by harm. Patients are considered first victims of patient safety events, while members of the workforce may experience a second victim impact, including physical and psychological distress ^5, 6^. How organisations respond to and learn from safety events affecting patients, families and staff is a marker of their patient safety culture and impacts the future safety and wellbeing of all concerned. 

A safety culture is about shared organisational beliefs, values, norms and procedures for patient safety ^7, 8^. Safety climate, on the other hand, relates to a group or team perception of safety culture in organisations ^9^. Rather than a specific process or technology, interventions to promote a culture of safety are designed to improve organisational conditions, enhancing leadership and teamwork among health care providers to support safe patient care ^10– 12^. Safety culture interventions which target staff behaviour include executive walk-rounds or interdisciplinary rounds; multicomponent unit-based interventions; team training or communication initiatives ^11^; frameworks for assessing, reporting and improving patient safety concerns; and standalone courses ^13, 14^. Patient safety educational interventions have also targeted the patient safety skills, attitudes and knowledge of healthcare workers to support safe patient care ^15^, with safety culture likely moderating their impact ^16^. Distinctions in terms and definitions are set out in Table 1.

**Table 1.** Definitions and terms.

Patient safety culture	One aspect of an organization’s culture. It can be personified by shared values, beliefs, norms, and procedures related to patient safety among members of an organization, unit, or team. It influences clinician and staff behaviours, attitudes, and cognitions through cues about the relative priority of patient safety compared to other goals such as throughput or efficiency ^11^.
Safety culture	The product of individual and group values, attitudes, perceptions, competencies, and patterns of behaviour that determine the commitment to, and the style and proficiency of, an organisation’s health and safety management ^4^.
Safety climate	Group or team perceptions of safety culture in organisations. These are measurable aspects of safety culture, in contrast to domains of culture such as behaviour and values ^9^.

Evidence on the effectiveness of interventions to improve safety culture is weak, but suggests they contribute to clinical care processes and to improved clinician and staff perceptions of safety culture ^11^. A safety culture can support staff to deliver effective and timely care to patients, increase staff competencies to collectively learn from patient safety events when they occur ^17^, and can strengthen organisational support to healthcare teams to recover from patient safety events ^18– 22^. Despite a focus on patient safety within measures of safety culture, there is inconsistent evidence for the link between interventions seeking to enhance a safety culture and patient outcomes such as hospital readmission rates, length of stay, pressure ulcers and falls, or ventilator associated pneumonia ^15, 23– 27^. The knowledge base on safety culture and patient outcomes is mixed, and a safety culture can moderate the effects of patient safety interventions on patients. 

Measures of safety culture focus on staff attitudes and behaviours related to the prioritisation of patient safety ^4, 28, 29^. In hospitals, evaluations of safety culture typically measure workforce related domains such as teamwork, communication, or information exchange, safety culture outcomes such as reporting rates, and patient outcomes such as falls, length of stay, or readmission rates ^11, 23^. A minority of measures include domains related to staff outcomes such as staff safety behaviours, staff injury rates, or well-being following a patient safety event ^30^. Reflecting the measurement focus of instruments, interventions to enhance safety culture are generally targeted at enhancing the norms and behaviours of hospital staff, yet, without an associated focus or exploration of the staff outcomes associated with safety culture. Evidence suggests that a safety culture can alleviate stress and burnout for staff ^31– 33^, support recovery and learning from events, and safeguard against repeat events ^34– 37^ by supporting staff to safely speak out about what is not working ^30, 38^. There is a now a need for a review of the impact of safety culture interventions on staff, to generate a common understanding of what staff outcomes are associated with interventions to improve safety culture and what staff outcomes should be measured. This research seeks to isolate staff outcomes, to examine the impact of safety culture interventions on staff where staff effects are claimed to take place.

Safety culture measurement tools focus on generating data to improve the safety of care provided to patients, and they do so by measuring staff perceptions of safety culture ^39^, including staff attitudes, behaviours, and norms ^40^. Depending on the instruments selected, staff outcomes and experiences may be captured through the deployment of safety culture measurement tools alone or in combination with other data sources. Prior research suggests a bi-directional relationship between safety culture and staff experience. The strongest evidence suggests that a safety culture influences certain staff outcomes such as staff behaviour to improve safety, event reporting, staff turnover, and injury rates among staff ^30, 41^. This literature reviews seeks to isolate staff outcomes as the point of interest including, but not limited to, burnout, stress, well-being, turnover or absenteeism ^32, 42^.

Hospital staff experiences of and outcomes from interventions to improve safety culture are rarely examined in their own right. There is a knowledge gap on how safety culture affects staff in hospital settings. This systematic review seeks to address the knowledge gap and generate a conceptual understanding of associated staff outcomes based on the available published evidence, evaluating the state of evidence connecting safety culture to staff outcomes. Understanding these issues is important: as the Lancet has stated, ‘there is no patient safety without health worker safety’ ^43^.

## Systematic review aims and objectives

A systematic review of the literature will examine the state of evidence connecting interventions to improve safety culture to hospital staff outcomes. The research questions are:

1) What effects do interventions to improve safety culture have on hospital staff?
2) What intervention features, safety culture domains or other factors explain these effects?
3) What staff outcomes and experiences are identified?

## Design

This study is a mixed methods systematic review ^44, 45^ of published literature guided by the Preferred Reporting Items for Systematic Reviews and Meta-Analyses (PRISMA) guidelines ^46^.

## Methods

### Search strategy

Searches will be conducted using the electronic databases of MEDLINE, EMBASE, CINAHL, Health Business Elite, and Scopus. The search strategy will include the following terms: patient safety culture, hospital workforce and management, hospital setting, and high-, middle- and low-income countries. The primary search will begin in PUBMED with major keywords listed on the Medline Medical Subject Headings (MeSH) terms, such as “patient safety,” “safety climate,” and “hospital workforce”. Search terms will be used in conjunction with Boolean Operators “AND” and “OR.” Supplementary Files will provide details of the full search strategy. Reference lists of all articles included for full-text screening will also be searched. A sample of the search run through Medline is provided in Table 2.

**Table 2.** Search strategy.

Keyword	Related terms/synonyms
Intervention	(safety OR quality OR organisation* OR just OR learning OR no-blame) N2 (culture OR climate OR attitude OR patient OR support OR management OR improvement OR enhancement) N2 (intervention OR initiative OR strategy) OR ((MH "Patient Safety") OR (MH "Safety+) AND (MH "Patients+")) AND ((MH "Quality Improvement+") OR (MH "Organizational Culture"))
Context	(health OR acute OR hospital) N2 (care OR center* OR centre* OR setting OR environment OR department OR division* OR facilit* OR team* OR unit*) OR (MH "Hospitals+")
Population	(administrat* OR allied OR auxiliary OR clinical OR frontline OR health* OR hospital OR licensed OR manager OR management OR medical OR nurs* OR operations OR social OR support OR trained OR welfare) N2 (aide* OR agent* OR assistant* OR attendant* OR auxiliar* OR carer* OR caregiver* OR consultant* OR distributor* OR individual* OR mentor* OR officer* OR person OR personnel OR practitioner* OR profession* OR professional* OR promotor* OR provider* OR service* OR staff OR surveyor* OR therap* OR worker*) OR (clinician* OR counselor* OR counsellor* OR doctor* OR doula* OR “general practitioner*” OR hospitalist* OR midwi* OR nurse* OR paraprofessional* N2 health* OR physician* OR physiotherapist OR psychotherapist* OR therapist* OR pharmacist*) OR (MH "Health Personnel+")
Outcome	performance OR outcome OR burnout OR stress OR satisfaction OR improv* OR well-being OR second victim OR report* OR working conditions OR behavior OR turnover OR injury rates OR problem solving OR safety competenc* OR recover* OR skill OR attitude OR knowledge OR trust OR learn*
*is used to signify truncation	

### Inclusion and exclusion criteria

Articles that report research involving both clinical and non-clinical members of the hospital workforce, and hospital management, will be included. Articles will be included if they are (a) quantitative, qualitative or mixed methods studies that evaluate an intervention in a hospital setting with an explicit aim to improve safety culture; (b) contain empirical data for analysis; (c) are available in any language; (d) are published in peer-reviewed scholarly journals; and (e) are published since 2000 (from the publication date of *To Err is Human*).

Articles will be excluded if they (a) describe interventions that do not have an explicit aim to improve safety culture, (b) do not measure the effect of the intervention on safety culture, and/or (c) have a primary purpose other than hospital staff or patient safety culture. Inclusion and exclusion criteria are illustrated in Table 3.

**Table 3.** Systematic review inclusion and exclusion criteria.

	Inclusion criteria	Exclusion criteria
Population	Hospital health care workers, including clinical, non-clinical and management staff.	Primary or Specialist Care
Intervention	Intervention studies designed to improve safety culture that include an explicit measure of safety culture.	Descriptions of safety culture interventions without any measures or outcomes captured.
Context	Hospitals Global: high-, middle-, and low-income country	Non-Hospital Settings
Outcome	Staff related safety culture outcomes and experiences that are identified following a hospital based intervention to improve safety culture.	Safety Culture Outcomes, Safety Climate Outcomes, or Patient Outcomes with no distinction of staff outcomes or experiences.
Date range	Published from 2000	Published before 2000
Publication Type	Research Article	Conference Abstracts, Conference Proceedings, Grey Literature, Reports
Languages	All	

### Article review process

Search results will be entered to Endnote where duplicates will be removed. Titles and abstracts of remaining articles will be screened for inclusion by two reviewers in Covidence, a systematic review data management program. Disagreement will be resolved by discussion with a third reviewer. Those selected for full-text review will be assessed according to the inclusion and criteria for addition to the final sample, again by two reviewers and discussion with a third to resolve disagreements.

### Data extraction and quality assessment

Data will be extracted from each article and organized into a matrix. The articles will be examined to determine if and how well they measure effect on or capture outcome experience of health care workers and management following safety culture interventions. Data will be organised according to author, year, sample size, sample characteristics, place of publication, study setting, study design, intervention structure, intervention content, intervention duration, outcomes measured, measurement instrument, and principal findings. Data from included articles will be assessed independently by two authors, and disagreements will be resolved by discussion until a consensus is reached. The mixed-methods appraisal tool (MMAT) will be used as a quality assessment tool, with two authors discussing and cross-checking quality scores ^46– 48^. The Cochrane Collaboration’s tool for assessing risk of bias in randomised trials ^49^ and non-randomised studies of interventions ^50^ will be employed to verify bias.

### Data synthesis and analysis

Synthesis will follow the Joanna Briggs Institute methodological guidance for mixed methods reviews, which recommends a convergent approach to synthesis and integration ^45^ following the work of Hong *et al*., ^47^ and Sandelowski ^48^. A convergent integrated approach, with simultaneous synthesis, is suitable as the review question can be answered by both quantitative and qualitative research designs, involves data transformation and allows reviewers to combine quantitative and qualitative data ^45^. To answer the research questions ‘what effects do interventions to improve safety culture have on staff?’; ‘what intervention features, safety culture domains or other factors explain these effects?’, and ‘what staff outcomes and experiences are identified?’ a thematic analysis of staff outcomes will be simultaneously combined with data on measures and effects available in the evidence. 

### Study status

Preliminary searches and study selection process piloted. We are currently formally screening search results against eligibility criteria.

## Data availability

No data are associated with this article. 
