# Supplementary material for: ‘What effect do safety culture interventions have on health care workers in hospital settings?’ A systematic review of the international literature
Source: HRB Open Res. 2022 Nov 18;5:48. Originally published 2022 Jul 1. [Version 2] doi: 10.12688/hrbopenres.13576.2 (PMC10357077; doi:10.12688/hrbopenres.13576.2)
Supplement: Peer review history

# Peer review: Reviewer response for version 2

**DOI:** 10.21956/hrbopenres.14928.r34743

**Published:** 2023-07-19

**Authors:** Ward Schrooten, Marlies Claesen

**Affiliations:** 1 Department of Medicine and Life Sciences, Hasselt University, Hasselt, Flanders, Belgium

The introduction provides different perspectives on the definition of safety culture and refers to the various domains of safety culture used in safety culture measurement tools. The focus of this systematic review is introduced.

 The aims and objectives of the study are clearly stated.

 The methods section explains the search strategy, inclusion and exclusion criteria, article review process, data extraction and quality assessment, and data synthesis and analysis. Tables 2 and 3 provide further detail.

 This study protocol describes an appropriate study design for the research question and sufficient detail to allow replication by others.

Is the study design appropriate for the research question?

Yes

Is the rationale for, and objectives of, the study clearly described?

Yes

Are sufficient details of the methods provided to allow replication by others?

Yes

Are the datasets clearly presented in a useable and accessible format?

Not applicable

Reviewer Expertise:

Outcome assessment, Clinical epidemiology, Quality and safety, Population health

We confirm that we have read this submission and believe that we have an appropriate level of expertise to confirm that it is of an acceptable scientific standard.

# Peer review: Reviewer response for version 2

**DOI:** 10.21956/hrbopenres.14928.r33209

**Published:** 2022-11-24

**Authors:** Laura Adamson

**Affiliations:** 1 Department of Radiation Oncology, Sydney West Radiation Oncology Network, Crown Princess Mary Cancer Centre, Sydney, NSW, Australia

No further comment.

Is the study design appropriate for the research question?

Yes

Is the rationale for, and objectives of, the study clearly described?

Yes

Are sufficient details of the methods provided to allow replication by others?

Yes

Are the datasets clearly presented in a useable and accessible format?

Not applicable

Reviewer Expertise:

Incident learning systems and safety culture in Radiation Oncology.

I confirm that I have read this submission and believe that I have an appropriate level of expertise to confirm that it is of an acceptable scientific standard.

# Peer review: Reviewer response for version 1

**DOI:** 10.21956/hrbopenres.14825.r33029

**Published:** 2022-10-18

**Authors:** Laura Adamson

**Affiliations:** 1 Department of Radiation Oncology, Sydney West Radiation Oncology Network, Crown Princess Mary Cancer Centre, Sydney, NSW, Australia

Thank you for the opportunity to review this protocol to perform a mixed methods systematic review on healthcare worker staff outcomes of safety culture interventions.

 The study protocol is clear. The rationale for investigating the gap in the literature is well stated and supported. I anticipate that there will be a variety of interventions, and safety culture assessments across the literature. 

 Some minor comments and recommendations:

- With multiple safety culture measurement tools within in the literature, the authors should ensure they note the disparity between the tools. Consideration will be needed when comparing the different assessment tools and the authors should avoid direct comparison. I wonder if any additional questions around what measurement tools of safety culture are currently used.
- The authors state that the Lancet has stated "xxx" in a sentence, however it is not the Lancet, but the author that has published in the Lancet, that has stated that this sentence should be corrected to Shaw *et al.* or similar stated. 

Is the study design appropriate for the research question?

Yes

Is the rationale for, and objectives of, the study clearly described?

Yes

Are sufficient details of the methods provided to allow replication by others?

Yes

Are the datasets clearly presented in a useable and accessible format?

Not applicable

Reviewer Expertise:

Incident learning systems and safety culture in Radiation Oncology.

I confirm that I have read this submission and believe that I have an appropriate level of expertise to confirm that it is of an acceptable scientific standard, however I have significant reservations, as outlined above.

# Response

**Published:** 2022-11-10

**Authors:** Mairéad Finn

**Affiliations:** Royal College of Surgeons in Ireland, Ireland

Thank you to both reviewers for the time and reflection taken to appraise this work. Their feedback greatly enhances the piece and we are grateful for the opportunity to add clarity and further investigate the literature. A short summary of our updates is: an enhanced review of the deployment of safety culture as a concept, and the differing perspectives therein; an incorporation of the new research question recommended; a clarification on the specific focus and limitation of the research; clarification on the treatment of varying measurement instruments in the review; and an adjustment to the reference in the Lancet, as observed by both reviewers.

 Laura Adamson:

 Thank you for these observations. We are collating findings as per the individual survey domains and then collating these to common high level narrative themes as developed by Churruca *et al.* ( https://bmjopen.bmj.com/content/11/7/e043982). This is not for direct comparison but rather to enable a high level integrated narrative on the studies included. Churruca *et al *used these successfully in a recent systematic review of tools. Please see below extract to make clear to reader:

 “A recent development has been the development by Churruca and colleagues of common over-arching thematic labels to bring together and describe the domains of safety culture across different assessment tools. While tools are not directly comparable, these common themes make high level comparsions more amenable.”

 Thank you also for noting the point on authorship in the Lancet publication: amended as detailed.

# Peer review: Reviewer response for version 1

**DOI:** 10.21956/hrbopenres.14825.r32559

**Published:** 2022-08-22

**Authors:** Jane K. O'Hara

**Affiliations:** 1 NIHR Yorkshire and Humber Patient Safety Translational Research Centre, Bradford, UK

Thank you for the opportunity to review this article. I found it generally clear and well argued. Whilst there have been many reviews on general safety culture, and patient safety culture, this does seem to add to this already large literature by focusing explicitly on the relationship between interventions and the impact on staff outcomes. I applaud the authors for taking on such a review, as I can imagine that actually working through the papers, with their likely vast array of different types of interventions and definitions of safety culture, will be a significant undertaking.

 I do have a few comments and suggestions for the authors to consider in their revisions.

  

- Is the rationale for, and objectives of, the study clearly described?
  The paper clearly describes a set of research questions. However, I do have a couple of queries for the authors on the scope of the questions. The term safety culture is contested within academia and practice. The authors describe some of this variation in the term in their rationale, but I think they could, and perhaps should reflect this a little more in the body of their introduction. This is particularly important given that there is an implicit assumption embedded in this type of review (exploring the impact of an intervention on an outcome) that the construct that is foundational to the intervention can be manipulated, therefore leading to some change in the outcome. In the context of safety culture, there are some authors that would argue that it cannot be manipulated, but rather is an emergent property of a system – an outcome of other processes and structures – or at the very least, bidirectional (as the authors themselves mention). Of course, we can’t be sure what position is ‘right’ but I do think the authors need to be clear that the manipulation of culture is in itself contested, and that by doing the review, they are taking an explicit position on this discussion. A good commentator on this issue is Stian Antonsen - I would urge the authors to engage in his work in this paper and the subsequent final review.
  In terms of the research questions, because of this disagreement within the field I wonder if the review would benefit from an explicit research question about the nature of the definition of safety culture contained within studies looking to manipulate it?
- Is the study design appropriate for the research question?
  Largely, I think the design is appropriate. However, I do have a slight concern about the exclusion of research that does not present an explicit measure of safety culture as part of the intervention. This would exclude papers reporting research that is based broadly on the position that improving safety culture or facets of safety culture (including all of the varied ways that culture is conceived – for example, team communications and team working) might lead to improvements in an outcome of interest, but that don’t undertake measurement of ‘safety culture’. This in and of itself is not necessarily a problem, but I think the authors need to be clear the impact this will have on the review and the findings. It is also likely to exclude most qualitative work, which from my reading of the methods is included in the review.
- Are sufficient details of the methods provided to allow replication by others?
  Yes.
- Are the datasets clearly presented in a useable and accessible format?
  The databases to be reviewed are stated clearly.

**General comments:**

- Just a small, perhaps pedantic, point. At the end of the rationale, the authors state:
  “ *Understanding these issues is important: as the Lancet has stated, ‘there is no patient safety without health worker safety’ ^43^*."
  The Lancet did not state this; rather, the authors who were published in The Lancet said that within that particular paper.
- There are a couple of typographical errors in the manuscript – perhaps editorial could attend to these?

Is the study design appropriate for the research question?

Partly

Is the rationale for, and objectives of, the study clearly described?

Partly

Are sufficient details of the methods provided to allow replication by others?

Yes

Are the datasets clearly presented in a useable and accessible format?

Yes

Reviewer Expertise:

NA

I confirm that I have read this submission and believe that I have an appropriate level of expertise to confirm that it is of an acceptable scientific standard, however I have significant reservations, as outlined above.

# Response

**Published:** 2022-11-10

**Authors:** Mairéad Finn

**Affiliations:** Royal College of Surgeons in Ireland, Ireland

Thank you to both reviewers for the time and reflection taken to appraise this work. Their feedback greatly enhances the piece and we are grateful for the opportunity to add clarity and further investigate the literature. A short summary of our updates is: an enhanced review of the deployment of safety culture as a concept, and the differing perspectives therein; an incorporation of the new research question recommended; a clarification on the specific focus and limitation of the research; clarification on the treatment of varying measurement instruments in the review; and an adjustment to the reference in the Lancet, as observed by both reviewers.

 Jane O'Hara:

 Thank you for these welcome observations. Drawing on the work of Stian Antonsen, in our introduction we have further developed varying theoretical approaches to the understanding and investigation of safety culture. We draw attention to differing approaches and acknowledge the specific focus of our research question relates to studies that view safety culture as manipulable (see also inclusion and exclusion criteria). We will clarify that no one position is right and be clear that the manipulation of culture is in itself contested, and that by doing the review, we are focusing on studies that take an explicit position on this discussion. Our position aligns to studies that seek to measure changes to culture in a system, though we do not argue this is ‘right’.

 Furthermore, we have since conducted the search and know now the variations in scale and time frame of included studies. For example, one study conducted a 3-year long intervention to influence culture. Several interventions were multi-pronged across long time frames. We will discuss these findings in the final review. 

 We have added a research question on the definitions of safety culture contained within the included studies: “How is safety culture defined in studies with interventions that aim to enhance it.” We will record these and any theory of change documented in the included studies.

 We have made both the explicit focus and limitation of our study more clear to readers. In the section on inclusion and exclusion criteria, we include the statement: “From a theoretical perspective, the research approach may limit the review to studies that view safety culture as amenable to change within a healthcare system, and exclude conceptual studies. The approach adopted is an explicit focus limited to studies that seek to measure change in safety culture as a totality and not sub-elements of safety-culture only. Limitations of the study design, such as bias toward quantitative studies and interventions which target safety culture will be acknowledged in the findings.”

 Thank you for identifying the correct authorship within the Lancet publication, we will amend.
